# Influence of Titanium Surface Residual Stresses on Osteoblastic Response and Bacteria Colonization

**DOI:** 10.3390/ma17071626

**Published:** 2024-04-02

**Authors:** Rita Pereira, Paulo Maia, Jose Vicente Rios-Santos, Mariano Herrero-Climent, Blanca Rios-Carrasco, Conrado Aparicio, Javier Gil

**Affiliations:** 1 Facultad de Odontología, Universidad de Sevilla, Calle Avicena s/n, 41009 Sevilla, Spain; aritacpereira@gmail.com (R.P.); jvrios@us.es (J.V.R.-S.); brios@us.es (B.R.-C.); 2 Facultade Ciências da Saúde, Universidad Europeia de Lisboa,1500-210 Lisboa, Portugal; paulormaia@gmail.com; 3 Porto Dental Institute, 4150-518 Porto, Portugal; dr.herrero@herrerocliment.com; 4 Facultad de Odontología, Universitat Internacional de Catalunya, c/ Josep Trueta s/n, 08195 Sant Cugat del Vallés, Spain; cjaparicio@uic.es; 5 Bioengineering Institute of Technology, Universidad Internacional de Catalunya, c/ Josep Trueta s/n, 08195 Sant Cugat del Vallés, Spain

**Keywords:** titanium, grit blasting, dental implants, residual stress, osteoblasts

## Abstract

Grit basting is the most common process applied to titanium dental implants to give them a roughness that favors bone colonization. There are numerous studies on the influence of roughness on osseointegration, but the influence of the compressive residual stress associated with this treatment on biological behavior has not been determined. For this purpose, four types of surfaces have been studied using 60 titanium discs: smooth, smooth with residual stress, rough without stress, and rough with residual stress. Roughness was studied by optic interferometry; wettability and surface energy (polar and dispersive components) by contact angle equipment using three solvents; and residual stresses by Bragg–Bentano X-ray diffraction. The adhesion and alkaline phosphatase (ALP) levels on the different surfaces were studied using Saos-2 osteoblastic cultures. The bacterial strains Streptococcus sanguinis and Lactobacillus salivarius were cultured on different surfaces, determining the adhesion. The results showed that residual stresses lead to increased hydrophilicity on the surfaces, as well as an increase in surface energy, especially on the polar component. From the culture results, higher adhesion and higher ALP levels were observed in the discs with residual stresses when compared between smooth and roughened discs. It was also found that roughness was the property that mostly influenced osteoblasts’ response. Bacteria colonize rough surfaces better than smooth surfaces, but no changes are observed due to residual surface tension.

## 1. Introduction

Dental implants represent a valid therapeutic option for the replacement of missing teeth [1]. The development of implantology has made it possible to broaden the scope of dental treatment in that, through the placement of an implant, it is possible to provide long-term stable support for a dental prosthesis subjected to masticatory load [1,2]. The biological principles underlying the functioning of implants have been described by several authors and are summarized in the concept of osseointegration. Osseointegration is defined as the direct and structural connection of living and ordered bone with the surface of an implant subjected to a functional load [3]. The first studies on this phenomenon were carried out by Branemark in the 50s, 60s, and 70s [4,5], and in parallel by Schröeder [6,7,8], who were able to demonstrate that the alveolar bone is able to form a direct connection with an alloplastic material such as titanium in the form of a screw, once placed in a surgically created bed [9,10,11]. Implant surface characteristics have been shown to influence bone healing of the surrounding bone [2] and it has been histologically demonstrated that osseointegration can be achieved in a time range of six weeks under normal conditions with rough surfaces [11].

The morphology of these surfaces is involved in a number of biological events that occur after implant placement, ranging from protein adhesion to peri-implant bone remodeling. These phenomena are favored by a certain surface roughness allowing a faster osseointegration which, from the clinical point of view, leaves room for the possibility of placing the prosthesis in shorter times. Usually, dental implants have roughened surfaces to optimize the processes of adhesion, proliferation, and osteoblastic differentiation, with the aim of generating bone around the dental implant and obtaining a mechanical and biological fixation [12,13]. The most common method of obtaining this topography is by grit blasting, in which abrasive particles, usually aluminum oxide, are projected to create a surface with a specific roughness. As is well known, osteoblastic cells are sensitive to roughness between 1 and 2 μm of Sa, and these values can be achieved by optimizing factors such as the particle projection pressure, distance from the projection gun to the surface, and size of the abrasive particles, among others [14,15]. Roughness optimization studies should also consider the affinity of bacteria for roughness. It is well known that increases in roughness favor bacterial adhesion and favor the creation of biofilms that can lead to peri-implantitis disease [16]. For this reason, roughness values have been adjusted to Sa between 0.7 to 1.2 μm, so that they provide a balanced roughness topography that favors osteoblastic response without notably increasing bacterial colonization [17,18].

Many studies have studied the influence of surface roughness on the biological response in vitro and in vivo, but the influence of surface compressive residual stresses as a result of grit particle projection has not been determined yet. For this purpose, smooth and rough titanium without and with residual stresses have been studied here. The hypothesis of this research is that the residual surface tension generated by the grit blasting treatment used to roughen the surface enhances osteoblastic cellular and microbiological activity. This hypothesis is based on the fact that the residual stress causes an increased hydrophilicity on the surface of the dental implant, favoring protein adsorption and facilitating osteoblastic cell adhesion as well as bacterial colonization.

## 2. Materials and Methods

### 2.1. Materials

Eighty discs of commercially pure titanium (grade 3) (Klockner Dental Implants, Escaldes-Engordany, Andorra) were tested and divided into four groups:

- Smooth titanium with no residual stress (S);
- Smooth titanium with residual stress (S + RS);
- Rough titanium without residual stress (R);
- Rough titanium with residual stress (R + RS).

Twenty original smooth discs formed the first studio group (S). Another twenty smooth discs were subjected to a compression test, reaching a compressive stress. The mechanical tests were performed with a Bionix MTS electromechanical machine (MTS, Minneapolis, MN, USA) (S + RS). The crosshead speed was 1 mm/min.

The other 40 discs were shot-blasted with 600 micrometer-sized alumina at a distance of 100 mm with a pressure of 2.5 bar for 120 s. The discs obtained showed roughness and had a residual surface tension due to the projection of the abrasives (R + RS). Twenty of these discs were heat treated at a temperature of 800 °C for 30 min under vacuum with a vacuum furnace (Hobersal 6300XB, Caldes de Montbui, Spain) in order to remove the residual stress (R).

To determine the compressive stress to be applied to the smooth discs, the residual stress of these discs was first determined by X-ray diffraction (Bragg-Bentano) (Siemens, Munich, Germany), as will be explained in the following sections. Once this was known, mechanical compression tests were carried out on the smooth discs in order to obtain a residual stress very similar to that produced by grit blasting. The stress applied for this residual stress was 678 ± 35 MPa within the plastic field of the disc.

### 2.2. Roughness, Wettability, and Surface Energy

**Figure 1:**
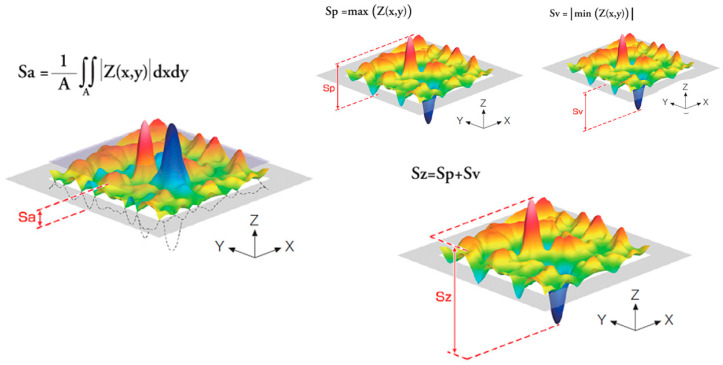
Roughness parameters scheme. Sa expresses, as an absolute value, the difference in height of each point compared to the arithmetical mean of the surface. This parameter is generally used to evaluate surface roughness. The maximum height Sz is equivalent to the sum of the maximum peak height Sp and maximum valley depth Sv. The colors in the scheme represent different heights. From the red color with the highest height to the blue color with the lowest depression.

White light interferometry (Wyko NT1100 Interferometer, Veeco Instruments, Plainview, NY, USA), in the vertical scanning interferometry mode, was used to produce, evaluate, and quantify the topographical features of the tested surfaces. The interferometric technique is ideal for imaging these surfaces as a large area of the surface can be imaged with a high vertical resolution (≈2 nm). The analysis area was 124.4 × 94.6 µm. Data analysis was performed with Wyko 32 (Veeco Instruments, USA), which allows the application of a Gaussian filter to separate waviness and form from roughness. Three different specimens of each type were measured to determine the amplitude parameter (Sa), the maximum peak value (Sz), and the hybrid parameter (index area) [13]. In Figure 1, the scheme of the different roughness parameters studied can be observed.

The contact angle analysis was performed on n = 3 samples with ultrapure distilled water (Millipore Milli-Q, Merck Millipore Corporation, Darmstadt, Germany) and formamide (Contact Angle System OCA15plus, Dataphysics, Filderstadt, Germany), and the corresponding data were analyzed with an SCA20 goniometer (Dataphysics, Filderstadt, Germany). Contact angle measurements were made using the sessile drop method. Drops were generated with a micrometric syringe and were deposited over discs. A total of 3 μL of distilled water and 1 μL of formamide were deposited on each sample at 200 μL/min. Finally, the surface free energy was determined by applying the Owens, Wendt, Rabel, and Kaelble (OWRK) equation, with wettability values obtained with distilled water and formamide, and the Wenzel equation for the correction of contact angles with the roughness [19].

### 2.3. Residual Stresses

Residual stresses were measured with a diffractometer incorporating a Bragg–Bentano configuration (D500, Siemens, Munich, Germany). The measurements were performed for the family of planes (213), which diffracts at 2θ = 139.5°. The elastic constants of Ti at the direction of this family of planes are EC = (E/1 + ν)(213) = 90.3 (1.4) GPa. Eleven Ψ angles, 0°, five positive, and five negative angles, were evaluated. The positions of the peaks were adjusted with a pseudo-Voigt function using appropriate software (WinplotR, version 2.1 free access on-line), and then converted to interplanar distances (dΨ) using Bragg’s equation. The d Ψ vs. sen2Ψ graphs and the calculation of the slope of the linear regression (A) were performed with appropriate software (Origin version 3.0, Microcal, Piscataway, NJ, USA). The residual stress is σ = EC(1/d_0_)A; where d_0_ is the interplanar distance for Ψ = 0° [20].

### 2.4. Osteoblast Culture

For in vitro studies, osteoblastic cells (Saos-2; ATCC, Manassas, VA, USA) were used. They were cultured in McCoy’s modified 5A medium, supplemented with 10% fetal bovine serum (FBS, Gibco, New York, NY, USA), 1% penicillin/streptomycin 2 mM (Invitrogen, Carlsbad, CA, USA), and 1% sodium pyruvate (Invitrogen, Carlsbad, CA, USA). Cultures were grown at 37 °C in a 5% CO_2_ incubator under humidified conditions, with n = 25 for each treatment.

Confluent cells were incubated with TrypLE (Invitrogen, Carlsbad, CA, USA) for 1 min to detach them from the flask. Subsequently, 5000 cells were seeded on each disc and incubated at 37 °C. After 3 and 21 days of incubation, the samples were washed with PBS and moved onto a new plate to perform the metabolic activity assay using Alamar Blue (Invitrogen-Thermo Fisher Scientific, Waltham, MA, USA), following the protocol. Briefly, the reagent was prepared and pipetted to cover the samples, and the percentage of Alamar Blue reduction was estimated after 4 h of incubation at 37 °C, using the Alamar Blue solution as a blank.

### 2.5. Mineralization of the Osteoblasts

To study the osteoblasts’ differentiation, the alkaline phosphatase (ALP) activity was determined by the Sensolyte pNPP alkaline phosphatase colorimetric assay (Anaspec, Fremont, CA, USA). The determination of ALP was performed at a wavelength of 495 nm, and the detection was carried out using a conventional ELx800 microplate reader (Bio-Tek Instruments, Inc., Winooski, VT, USA).

### 2.6. Bacterial Strains and Growth Conditions

The bacterial strains Streptococcus sanguinis CECT 480 and Lactobacillus salivarius (CECT 4063) (Colección Española de Cultivos Tipo, Valencia, Spain) were used in this study. Bacteria were cultured in Todd–Hewitt broth at 37 °C in a 5% CO_2_-enriched atmosphere.

### 2.7. Evaluation of the Adhesion of Bacteria

The surface energy of the bacteria was evaluated according to an adaptation of the microbial adhesion to solvents (MATS) test [10]. The MATS test is a simplified method to characterize the electron donor/electron acceptor properties of microbial cells which, in turn, is a good parameter to predict bacterial adhesion to solid surfaces in an aqueous environment [21].

Bacteria were harvested during the exponential growth phase by centrifugation at 4500× *g* for 15 min at 4 °C, washed with phosphate-buffered saline (PBS) 0.15 M, and finally, suspended in the same PBS buffer, and the optical density was measured at 550 nm (A_0_). Three different solvents were used for the MATS analysis: chloroform, hexane, and diethyl ether. For each solvent, 3 mL of the bacterial suspension was placed in 9 glass tubes (10 mm in diameter) and 400 μL of solvent was added (3 samples for each solvent); it was then incubated at room temperature for 10 min and mixed on a vortex for 60 s. The aqueous phase was removed after 15 min and its optical density at 550 nm was measured (A_1_). The average microbial adhesion to solvent was calculated as (1 − A_1_/A_0_) × 100.

Cp-Ti discs with 5 mm diameter and 2.5 mm thickness were used in this study. The discs were washed with 70% ethanol, acetone, and distilled water, dried at room temperature and sterilized in an autoclave. The quantification of bacterial attachment was performed with two normal inhabitants of the mouth: *S. sanguinis* (CECT 480) and *L. salivarius* (CECT 4063). The Cp-Ti discs were incubated with bacterial broth (specific for each strain) for 2 h at 37 °C and 5% CO_2_, washed with PBS, and detached in Ringer’s solution. The new bacterial suspension was seeded on solid medium (MRS for *L. salivarius* and Todd–Hewitt for *S. sanguinis*), incubated for 48 h at 37 °C, and finally, the number of colonies were counted. At the same time, the pH variation during the bacterial growth phase was measured.

### 2.8. Statistical Analysis

The number of samples used was obtained by an experimental sample size method. Statistical analysis was performed using the MiniTab version 17 software (Minitab Inc., Lock Haven, PA, USA). The Kruskal–Wallis and Mann–Whitney U non-parametric tests were used to compare the different conditions to each other. Statistical differences were considered with *p* < 0.001.

## 3. Results

Table 1 shows the roughness, wettability, surface free energy, and residual stress values of the different surfaces.

**Figure 2:**
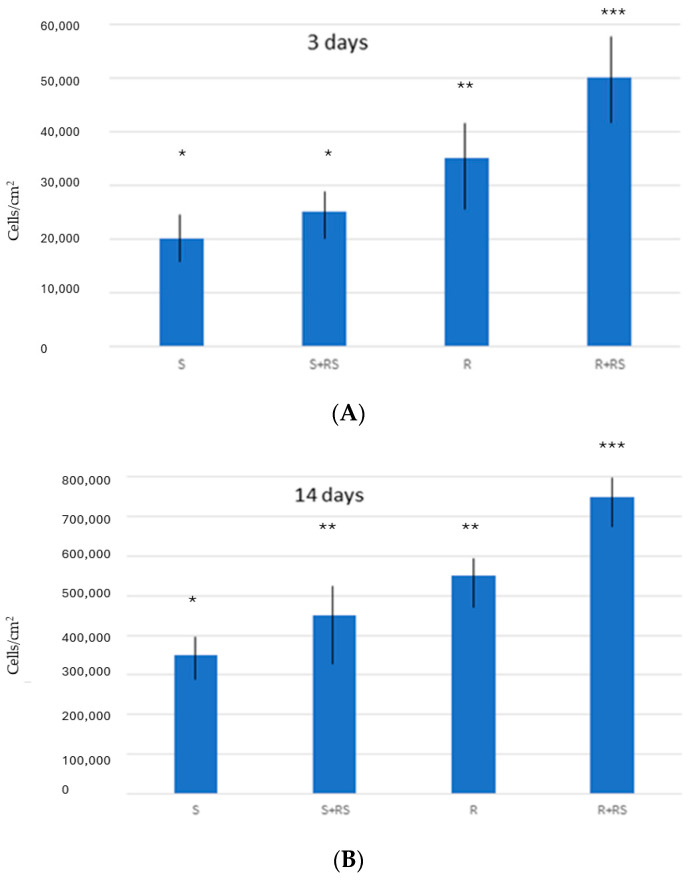
Proliferation of Saos-2 cells on surfaces: (**A**) after 3 days and (**B**) after 14 days of incubation. The results marked with one asterisk show statistically significant differences with respect to those marked with two asterisks and those marked with three asterisks show statistically significant differences with respect to those marked with two asterisks. The differences present at *p* < 0.001.

**Figure 3:**
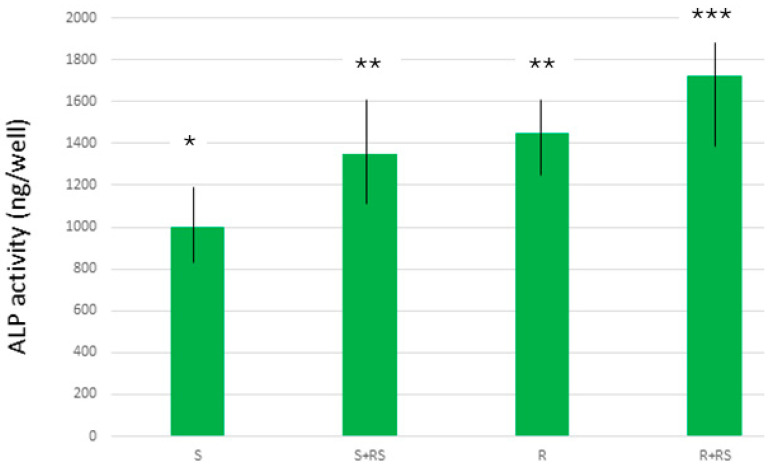
Production of ALP by Saos-2 cells on different surfaces. The results marked with one asterisk show statistically significant differences with respect to those marked with two asterisks and those marked with three asterisks show statistically significant differences with respect to those marked with two asterisks. The differences present at *p* < 0.001.

Figure 2 shows the number of osteoblastic cells on the different surfaces, showing a greater number on both smooth and rough surfaces where there is compressive residual stress, with statistically significant differences *p* < 0.001 for each of them. The same trend is also observed in the levels of ALP, showing a better osteoblast differentiation on surfaces with residual stress (Figure 3).

The results also clearly show that roughness favors cellular responses with respect to smooth surfaces. In fact, the topography of titanium surfaces affects the osteoblastic response towards bone regeneration the most.

**Figure 4:**
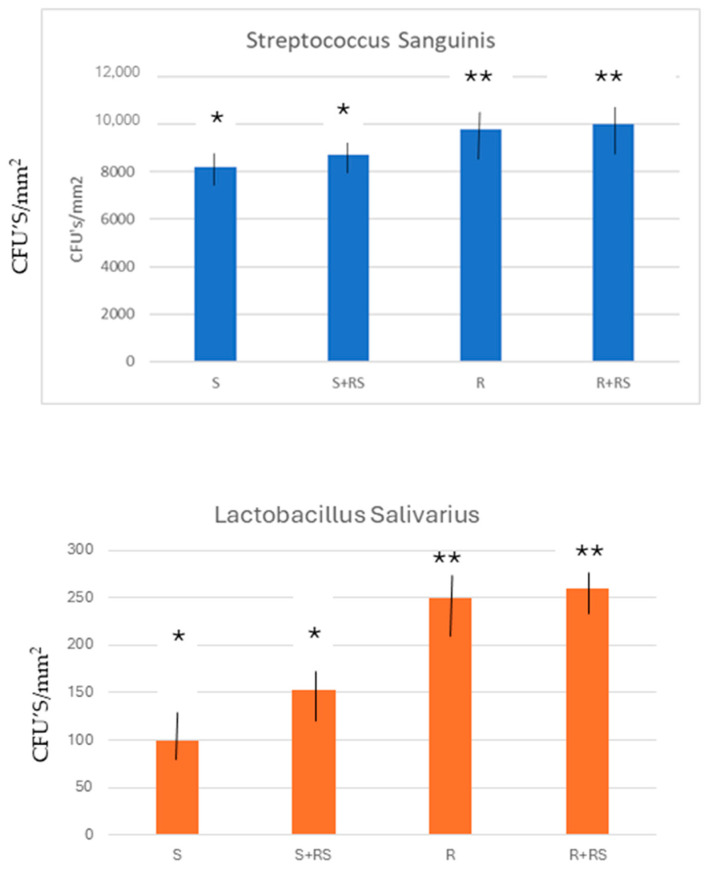
Bacterial colonies adhered on the different surfaces studied. Samples with the same symbol show no statistically significant differences between them. The results marked with one asterisk show statistically significant differences with respect to those marked with two asterisks. The differences present at *p* < 0.001.

The quantification of colony forming units (CFUs) per square millimeter (*p* < 0.001) can be observed in Figure 4; both strains showed a low trend in attaching to smooth (*L. salivarius*~1 × 10^2^/mm^2^, *S. sanguinis*~8.22 × 10^3^/mm^2^) compared to rougher surfaces.

The smooth samples with residual stress show values of 1.53 × 10^2^ for Lactobacillus salivarius and 8.70 × 10^3^ for Streptococcus sanguinis with respect to the values of 1.00 × 10^2^ and 8.22 × 10^3^, respectively. These results do not show statistically significant differences.

When we studied the influence of residual stress on the rough samples, a slight increase in colony formation by both bacteria was observed: from 2.20 × 10^2^ to 2.35 × 10^2^ for Lactobacillus salivarius and from 9.80 × 10^3^ to 10.32 × 10^3^ for Streptococcus sanguinis. However, the influence of residual stress does not present statistically significant differences with respect to the samples with no residual stress.

Statistically significant differences are seen when comparing colony forming values between smooth and rough samples, both on surfaces with residual stress and when comparing surfaces in the absence of residual stress.

The detached CFUs was quantified and compared in the same conditions (due to the real area in all samples not being equal). At the beginning the CFUs/mm^2^ quantifications in MRS and Todd–Hewitt broth showed a correlation with roughness (where rougher surfaces presented more CFUs/mm^2^ compared with smooth surfaces) possibly due to the influence of bacterial interactions associated with the roughness and wettability. We expect that this correlation could lead to initial bacteria attachment due to both strains presenting a hydrophilic trend, associated with weak interaction results [22,23,24,25].

## 4. Discussion

Table 1 shows that the smooth discs with residual stress have a lower contact angle than the original smooth ones, of approximately 20°, which indicates the surface becomes more hydrophilic. Similarly, when we compare the rough implants, we can see that the contribution of the residual stress is to improve wettability and increase surface energy, especially in its polar component.

Cells have the ability to adapt to the environment and surface characteristics. It is well known how osteoblasts prefer roughness between 1.5 and 3 μm Sa and the great capacity of fibroblasts to orient themselves in surface grooves. Pieuchot et al. [26] introduced the term “curvotaxis”, which allows cells to respond to curvature variations at the cellular scale. These authors elaborated sinusoidal surfaces with curvature modulations in all directions by determining cell activity in these topographies. It could be observed that cell adhesion avoids convex surfaces and preferentially adheres in concave areas. The authors show that curvotaxis depends on a dynamic interaction between the nucleus and the cytoskeleton, with the nucleus acting as a mechanical sensor that drives the migrating cell towards the concavity. Different authors have shown the sensitivity of the topographies of cell behavior and allow designs, such as curvotaxis, to control cell adhesion, proliferation, and subsequent mineralization by modifying the surfaces [27,28,29,30,31,32,33,34].

The higher hydrophilicity of the surfaces is very important for the interaction with the physiological environment, since the lower contact angle allows blood proteins to adsorb in greater quantities on the titanium surface. Surgeons observe two very important aspects for a good osseointegration prognosis. Firstly, if when drilling the bone they observe that there is abundant blood, this is a good sign for osseointegration; and the second aspect is the observation when placing the dental implant that the blood quickly covers the entire surface of the dental implant, i.e., when it has a very hydrophilic character [35].

Another property that modifies the residual stress of the titanium surface is the increase in surface energy, especially in the polar component. This fact also favors osteoblastic cell activity. Adsorption of proteins to surfaces is the first step in many fundamental biological processes, such as the blood coagulation cascade and transmembrane signaling.

There are multiple proteins in human blood plasma. These include fibrinogen (Fbg), fibronectin (Fn), and albumin (Alb). Fibronectin is a key component of the extracellular matrix (ECM), a large dimeric glycoprotein that triggers cell adhesion, assembles into cell-driven supramolecular fibrils, and provides specific binding sites for various ECM biopolymers. Pegueroles et al. [36] showed that the negative charge density on the titanium surface, or else the increase in the contribution of the polar component of surface energy, favors the adsorption of fibronectin, which is a precursor protein for osteoblastic cell-specific migration. Both the wettability and the polar component of the surface energy justify the good cell behavior and the high degrees of mineralization observed in the osteoblastic cell cultures.

In addition to improving the biological behavior, residual stress is known to improve the mechanical properties of dental implants. Several authors [28,29,30,31,32] have shown how compressive residual stress on the surface of the dental implant causes the inhibition of crack nucleation by cyclic chewing loads (fatigue). This is due to the fact that cracks are always formed by tensile stresses; if the surface is compressive it prevents crack formation. Scanning electron microscopy studies of dental implants subjected to fatigue show that in dental implants without compressive residual stress the crack is generated on the surface, and in implants with residual stress the crack nucleates approximately 20 μm from the surface. This nucleation requires much more damage to occur, which means many more load cycles on the implant and causes the implant to have a much higher fatigue limit [37].

**Figure 5:**
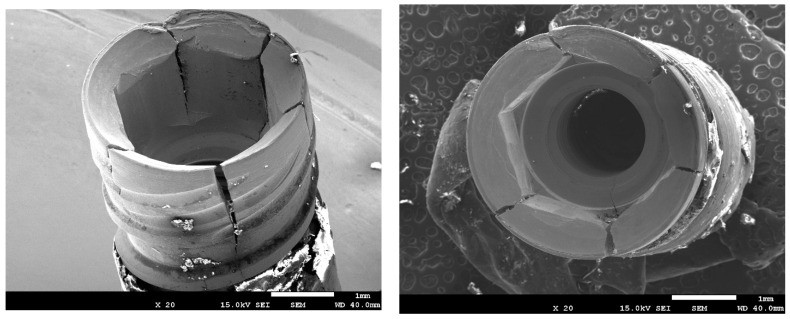
Fractures on the neck of the implant produced by fatigue in dental implants made of commercially pure titanium.

Currently, there has been an increase in the use of narrow dental implants to reduce, as much as possible, the destruction of bone and soft tissue of the patient. This fact causes dental implants to have a smaller section to support the physiological mechanical loads, producing an increase in fractures, as can be seen in Figure 5, where the dental implants fracture at the connection necks with the screw that joins the implant and abutment. Studies have been carried out with conventional implants and it has been determined that on many occasions conventional implants made of commercially pure titanium cannot withstand physiological stresses. Therefore, there are two ways to solve this situation: the manufacture of dental implants with an alloy that increases the maximum resistance of the implant, as is the case with the Roxolid^®^ implants of Straumann; or the manufacture of implants with Optimum^®^ titanium that are cold worked at 30%, such as those from Klockner Dental Implants. Bacteria colonize rough surfaces better than smooth surfaces, but no changes are observed due to residual surface tension. Currently, this last type of implant obtains better results since it produces a better osseointegration due to the compressive residual stress caused by the cold working. However, alloyed titanium increases the mechanical properties of the implant, but does not favor osteoblastic activity, as in the case of cold-worked implants [38,39,40].

Osteoblast cell culture studies show that cell proliferation values are higher in both smooth and rough osteoblast samples. This fact can be justified by the higher hydrophilicity values generated by surface tension [34,35,36]. Likewise, the surface energy values, and especially their polar component, causes the increases in the degree of proliferation and also in the levels of mineralization of the cells, as demonstrated by the higher levels of alkaline phosphatase [36]. The favoring effect of residual stress is seen in both types of surfaces. However, when comparing the results between smooth and rough surfaces it can be seen that the main role for cell activity is topography.

Biomineralization is the process by which hydroxyapatite is deposited in the extracellular matrix. The first step of mineralization is the formation of hydroxyapatite crystals within matrix vesicles that bud from the surface membrane of hypertrophic chondrocytes, osteoblasts, and odontoblasts. The hydroxyapatite then propagates into the extracellular matrix and is deposited between collagen fibrils. Extracellular inorganic pyrophosphate, provided by NPP1 and ANKH, inhibits hydroxyapatite formation [41,42].

ALP increases local inorganic phosphate rates and facilitates mineralization, in addition to reducing the extracellular pyrophosphate concentration. Among several isoforms, the non-tissue-specific isoenzyme of ALP (TNAP) is strongly expressed in bone, liver, and kidney and plays a key role in bone calcification. TNAP hydrolyzes pyrophosphate and supplies inorganic phosphate to enhance mineralization. The biochemical substrates of TNAP are believed to be inorganic pyrophosphate and pyridoxal phosphate. These substrates are concentrated under conditions of NAPT deficiency, resulting in hypophosphatasia. Increasing the level of ALP expression and development in this setting would undoubtedly provide new and essential information on the fundamental molecular mechanisms of bone formation and offer therapeutic possibilities for the treatment of bone-related diseases [43].

Alkaline phosphatase has sometimes been confused with osteocalcin. Alkaline phosphatase is an immediate indicator of mineralization processes, whereas the role of osteocalcin is more complicated but effects on osteoblastic differentiation have been attributed to it. Over the past few years, a great deal of research has been devoted to some sometimes controversial theories about osteocalcin (OCN). OCN is a 46-amino acid protein produced and secreted almost exclusively by osteoblasts, terminally differentiated cells responsible for the synthesis and mineralization of bone matrix during skeletal development and its periodic regeneration throughout life. Osteoblasts originate from mesenchymal progenitors and are short-lived cells that are constantly replaced, depending on the need for bone formation [44,45]. OCN, secreted by osteoblasts, contains three γ-carboxyglutamic acid residues that confer a high affinity for the hydroxyapatite bone matrix. However, when bone is resorbed by osteoclasts, a cell type derived from macrophages, the acidic pH of the resorption compartment causes the removal of the carboxyl groups of OCN, and the decarboxylated OCN is released into the circulation [46,47,48,49,50]. Circulating levels of decarboxylated OCN, thus, depend on the rate of bone turnover, also known as remodeling [51,52,53].

Initially thought to be an inhibitor of bone mineralization, recent studies suggest a broader role for osteocalcin that extends to the regulation of whole-body metabolism, reproduction, and cognition. Osteoblasts are specialized mesenchymal cells primarily responsible for the synthesis and deposition of the collagen-rich mineralized matrix that makes up bone tissue. In the last decade, studies have elaborated an expanded biological role for the osteoblast that focuses on the actions of bone-derived osteocalcin [49,50]. Osteocalcin has been commonly used as a serum marker of osteoblast bone formation and is thought to act in the bone matrix to regulate mineralization [54,55,56,57].

Bone is built by a process of self-assembly under the control of a gene regulatory network (GRN). Chekroun et al. [58] studied the behavior of bone gene regulation as a control of mineralization using mathematical equations. These authors estimated direct interactions between genes encoding transcription factors and those encoding bone proteins, as well as performing mathematical modeling of the bone GRN using a system of nonlinear differential equations modeling the interactions. The authors showed negative indirect interactions from negative feedback loops or micro-RNAs. These authors showed theoretical evidence of osteoblast self-inhibition following activation of the genetic regulatory network controlling mineralization. Bone metabolism is very complicated and further research is still needed to improve understanding.

With respect to the bacterial colonies studied, no significant differences were observed when comparing the presence or absence of residual stress on both surfaces. Although it could be thought that the variation in wettability would favor bacterial adhesion to hydrophilic or hydrophobic surfaces, depending on the character of the bacterial membrane, this could not be observed in this case, where Streptococcus has a more hydrophobic membrane and Lactobacillus more hydrophobic. It is possible that the difference in hydrophilic/hydrophobic character caused by residual stress is not sufficient to observe behavioral changes at the bacterial level but is sufficient for osteoblastic cells. The influence of surface energy and its two components, dispersive and polar, on bacterial adhesion should be further studied. It would be necessary to know the physicochemical properties of bacterial membranes in order to determine the mechanisms of adsorption and microbiological colonization.

What has been observed is that rough surfaces cause an increase in bacterial colony formation in both types of strains. This fact is common and has been observed by several authors and is a problem in dental implants since a rough surface is necessary for the improvement in osteoblastic activity and mechanical fixation of implant–bone tissue, but it also presents worse behavior towards bacteria and facilitates the formation of peri-implantitis.

With these results we can confirm the fulfillment of a part of the hypothesis, since we have been able to verify how residual stress favors osteoblast activity with the number of cells as well as osteocalcin levels in mineralization. However, the part of the hypothesis in which residual stress favors bacterial proliferation is not fulfilled. At this point it was only possible to verify that roughness favors the adhesion of the bacterial strains studied, but no significant differences were observed with the residual surface stress.

This manuscript is of clinical importance because in the placement of dental implants by clinicians, especially in narrow dental implants, the good properties, not only mechanical but also cellular activity, of dental implants obtained by cold working can be verified. The results of this study also give clinicians confidence that there is no increase in bacterial colonization caused by residual surface stresses.

## 5. Conclusions

It was determined that the compressive residual stress on the surface of both smooth and rough titanium improves the hydrophilicity and increases the surface energy of titanium, especially in its polar component. This fact causes osteoblastic proliferation to improve at 7 and 14 days of culture. It was observed that the main factor was roughness, since rough implants without residual stress showed a higher proliferation and degree of mineralization than smooth surfaces with residual stress. However, residual stress on smooth and rough surfaces caused proliferation and osteocalcin levels to be higher, with statistically significant differences. An increase in bacterial colonization was observed on rough surfaces compared to smooth surfaces, but the influence of residual stress on bacterial behavior could not be demonstrated.

## Figures and Tables

**Table 1:** Roughness parameters Sa, Sz, and index area, contact angle (CA), dispersive component of the surface energy (DC), polar component (PC), total surface energy (SFE), and residual stress (σ_residual_). The results marked with one asterisk show statistically significant differences with respect to those marked with two asterisks and those marked with three asterisks show statistically significant differences with respect to those marked with two asterisks. The differences present at *p* < 0.001.

	Sa (µm)	Sz (µm)	Index Area	CA (°)	DC (mJ/m^2^)	PC (mJ/m^2^)	Total SFE (mJ/m^2^)	σ_residual_ (MPa)
S	0.21 ± 0.02 *	0.34 ± 0.02 *	1.09 ± 0.01 *	77 ± 5 *	24.8 ± 1.2 *	10.2 ± 2.0 *	35.0 ± 3.2 *	−10 ± 2 *
S + RS	0.24 ± 0.10 *	0.41 ± 0.11 *	1.08 ± 0.06 *	58 ± 3 **	27.2 ± 1.2 **	18.3 ± 1.8 **	45.5 ± 2.2 **	−189 ± 20 **
R	2.04 ± 0.15 **	4.67 ± 1.07 **	1.66 ± 0.04 **	69 ± 4 *	27.7 ± 1.3 **	12.5 ± 2.1 *	40.2 ± 1.2 ***	−8 ± 3 *
R + RS	1.99 ± 0.18 **	4.89 ± 1.67 **	1.76 ± 0.04 **	53 ± 2 **	29.0 ± 2.2 **	20.4 ± 1.9 **	49.4 ± 1.8 **	−201 ± 12 **

## Data Availability

The authors can provide details of the research requirements by letter and comments if needed.
